# Dielectric breakdown of 2D muscovite mica

**DOI:** 10.1038/s41598-022-18320-7

**Published:** 2022-08-18

**Authors:** Anirudh Maruvada, Kalya Shubhakar, Nagarajan Raghavan, Kin Leong Pey, Sean J. O’Shea

**Affiliations:** 1 Engineering Product Development, Singapore University of Technology and Design, 8 Somapah Road, Singapore, 487372 Singapore; 2 Institute of Materials Research and Engineering, Agency for Science Technology and Research, 2 Fusionopolis Way, Singapore, 138634 Singapore

**Keywords:** Electrical and electronic engineering, Two-dimensional materials, Electronic properties and materials, Electronic devices, Information storage

## Abstract

Localized electrical breakdown (BD) measurements are performed on 2D muscovite mica flakes of ~ 2 to 15 nm thickness using Conduction Atomic Force Microscopy (CAFM). To obtain robust BD data by CAFM, the probed locations are spaced sufficiently far apart (> 1 µm) to avoid mutual interference and the maximum current is set to a low value (< 1 nA) to ensure severe damage does not occur to the sample. The analyses reveals that 2D muscovite mica has high electrical breakdown strength (12 MV/cm or more) and low leakage current, comparable to 2D hexagonal boron nitride (h-BN) of similar thickness. However, a significant difference compared to h-BN is the very low current necessary to avoid catastrophic damage during the BD event, even for very thin (2–3 nm) flakes. Further, for mica the BD transient always appear to be very abrupt, and no progressive BD process was definitively observed. These marked differences between mica and h-BN are attributed to the poor thermal conductivity of mica.

## Introduction

The two key drivers of the semiconductor industry are high-performance computing devices and high-density memory devices. Both applications heavily rely on the field-effect transistor (FET) which plays a key role in processing information in the case of computing devices and storing logic in the case of memory devices. The FET requires an ultra-thin insulating material, called the gate dielectric, across which electric fields are applied to tune the current flow in the transistor. At present amorphous oxides such as SiO_2_ and HfO_2_ commonly serve as the gate dielectric but limitations in the performance of these materials is being encountered as the FET dimensions are scaled down to optimize device performance^1^. As a consequence, considerable research has recently focused on using 2D materials as an alternative FET technology^2^ to enable continued miniaturization and long-term performance gains. In addition, there are growing new applications for 2D electronic materials in the fields of neuromorphic computing, quantum devices and photonic integrated circuits^3^.

An essential material required for a 2D FET is a compatible 2D insulator, which must have high dielectric constant and large effective insulator thickness. To date, hexagonal boron nitride (h-BN) has been considered almost exclusively for use as the 2D insulator^4^. An alternative but far less studied candidate material is 2D mica^5^. Mica is a van-der-Waals mineral whose individual layers can be exfoliated to form a defect-free, atomically flat surface^6,7^. Several properties potentially make mica an excellent candidate for a 2D insulator, namely its wide band gap with a direct band gap of 5.09 eV^8^, moderately high dielectric constant of 9.4^9^, and high thermal and chemical stability^10^.

Mica has been used as an insulating substrate for various heterogeneous 2D material structures, including mica/MoS_2_, mica/graphene, mica/graphene oxide, and mica/topological insulator configurations^11–20^. Of particular interest is the utility of mica in 2D microelectronic devices, and in this regard several recent studies have used mica as an active material in a resistive random memory (RRAM) device^21^ and as the gate insulator in a field effect transistor (FET)^12,13,17–19^. In all these microelectronic technologies, understanding the dielectric breakdown (BD) of the insulator material is critical to the reliability of the logic or data storage device. However, the BD mechanism of 2D mica has received little attention. Previous studies have been limited to mica ≥ 20 nm thick^22,23^, and there is only a single graph showing BD data on a 9 nm thick 2D mica sample^13^.

In this work, we investigate the dielectric breakdown of thin 2D mica flakes by local electrical stressing using conduction atomic force microscopy (CAFM). The results are compared with other 2D materials, namely synthetic mica and 2D hexagonal boron nitride (h-BN), to assess its candidacy as a prospective 2D insulator. We find that although the leakage current is low for both muscovite and synthetic mica, comparable to that of h-BN, breakdown occurs at very low current levels (~ 1 nA or less) even for 2–3 layer mica flakes. In comparison, current levels of ~ 100 nA or more are observed for the breakdown of h-BN and we attribute this contrast to the marked difference in thermal conductivity of the two materials.

**Figure 1:**
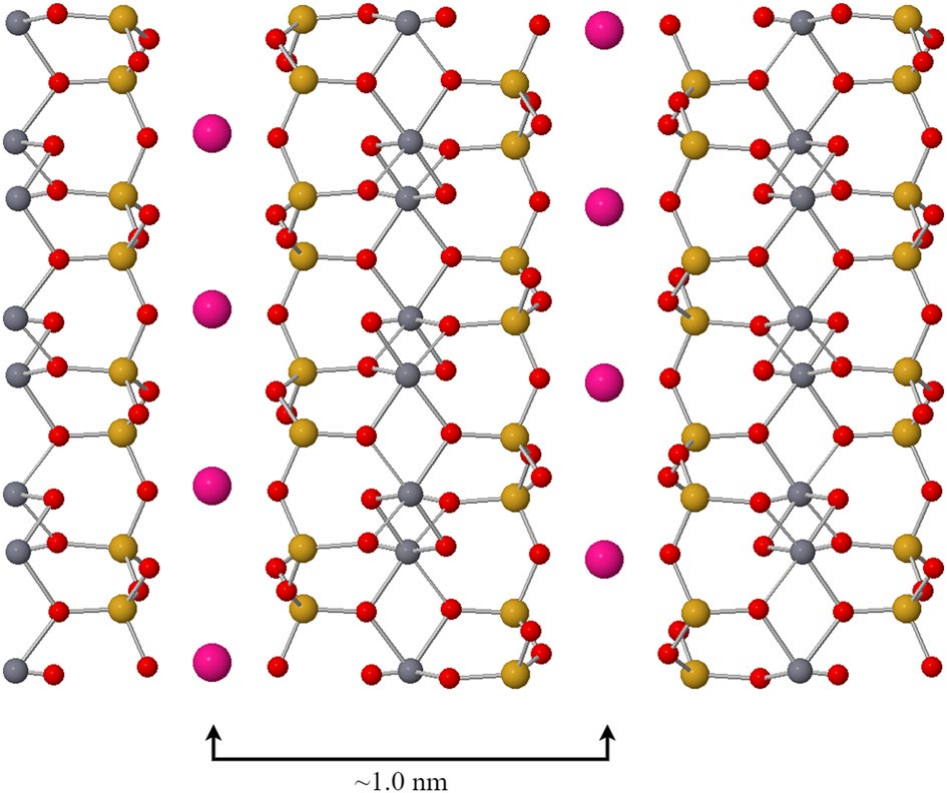
Model of the crystal structure of muscovite mica. The elements are colour coded Red = Oxygen, Grey = Aluminium, Pink = Potassium and Yellow = Silicon. Each mica sheet consists of an aluminium octahedron layer (aluminium, oxygen, hydrogen) sandwiched between two tetrahedron layers (silicon, aluminium, oxygen). Three sheets of mica are shown and potassium ions intercalate in-between these sheets. The mica cleaves readily between the potassium ion planes yielding a step height on exfoliation of 1.0 nm. The model is generated based on the reported literature^24^.

Before describing the experiments, a brief summary of the mica structure is appropriate. The most commonly studied mica is muscovite mica (KAl_2_(Si_3_AlO_10_)(OH)_2_), a naturally occurring material, whose crystal structure consists of an aluminium octahedron sandwiched between two silicon tetrahedron, as shown in Fig. 1^24^. The silicon tetrahedron layer has a partial substitution of silicon with aluminium to give it a negative charge^24,25^. Due to this, the positively charged potassium ions (K^+^) reside between the mica sheets to compensate for the charge. The mica sheets are held together by van der Waals forces, with one layer being around 1 nm thick, and can be exfoliated using different methods to form clean atomically flat 2D surfaces^6,7,26^. There are other types of natural mica with differing metal atoms (Fe, Ti, etc.) in the atomic structure. However, for application purposes, synthetic (or fluorphlogopite) mica appears more promising because it is more stable and can be heated to ~ 1100 °C with no degradation as compared to ~ 550 °C for natural mica variants^27^. Synthetic mica has a similar structure to natural muscovite mica (KMg_3_AlSi_3_O_10_F_2_) but the hydroxyl group is replaced by a fluoro group. Note that in our work, all the data presented is taken on muscovite natural mica, with the exception of one set of experiments undertaken on Fluorophlogopite synthetic mica shown in Supplementary Fig. S5.

## Results

### Experimental considerations when measuring 2D mica breakdown

**Figure 2:**
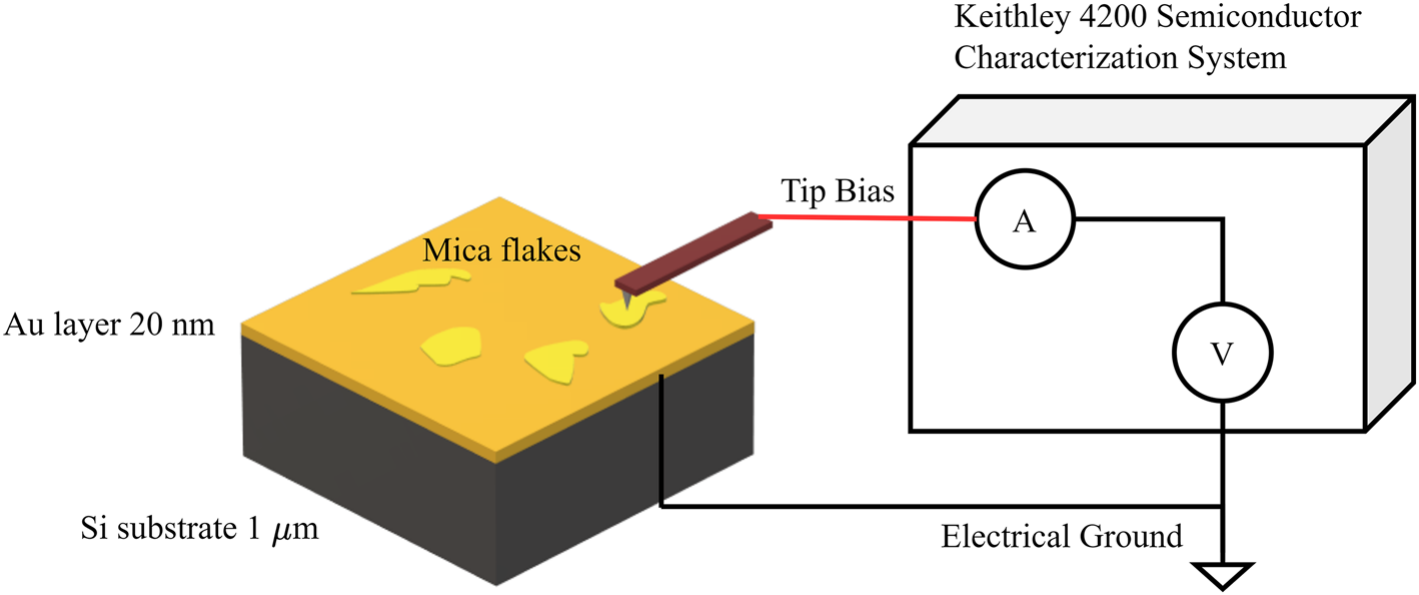
Schematic of the CAFM Setup. Mica flakes exfoliated on a Au substrate which acts as an electrode along with the AFM conducting tip. The electrodes are connected to the Keithley 4200 Semiconductor Characterization System with the substrate grounded.

We use gold assisted mechanical exfoliation of mica, as shown in Supplementary Fig. S1, to obtain thin (< 15 nm) and large (> 4 × 4 um^2^) mica flakes on a gold substrate^28–30^. CAFM is used to perform localized breakdown (BD) on the flakes by ramp voltage stress (RVS). In RVS, a voltage is linearly applied across the sample at a specific ramp rate until a defined current value (also called compliance current) is reached, at which time, the voltage is turned off to avoid further current injection into the sample. In our setup (shown in Fig. 2), a Keithley 4200 Semiconductor Characterization System is used to force voltage onto the tip and measure the current passing through the tip. The CAFM method operates with a conducting AFM tip in contact with the surface and enables localized BD and current-voltage (*I-V*) measurements to be made at any location on the surface. In our work, two types of conducting AFM tips are used, namely Platinum Iridium wire cantilevers and conducting diamond cantilevers, with the results being identical for both cantilever types.

**Figure 3:**
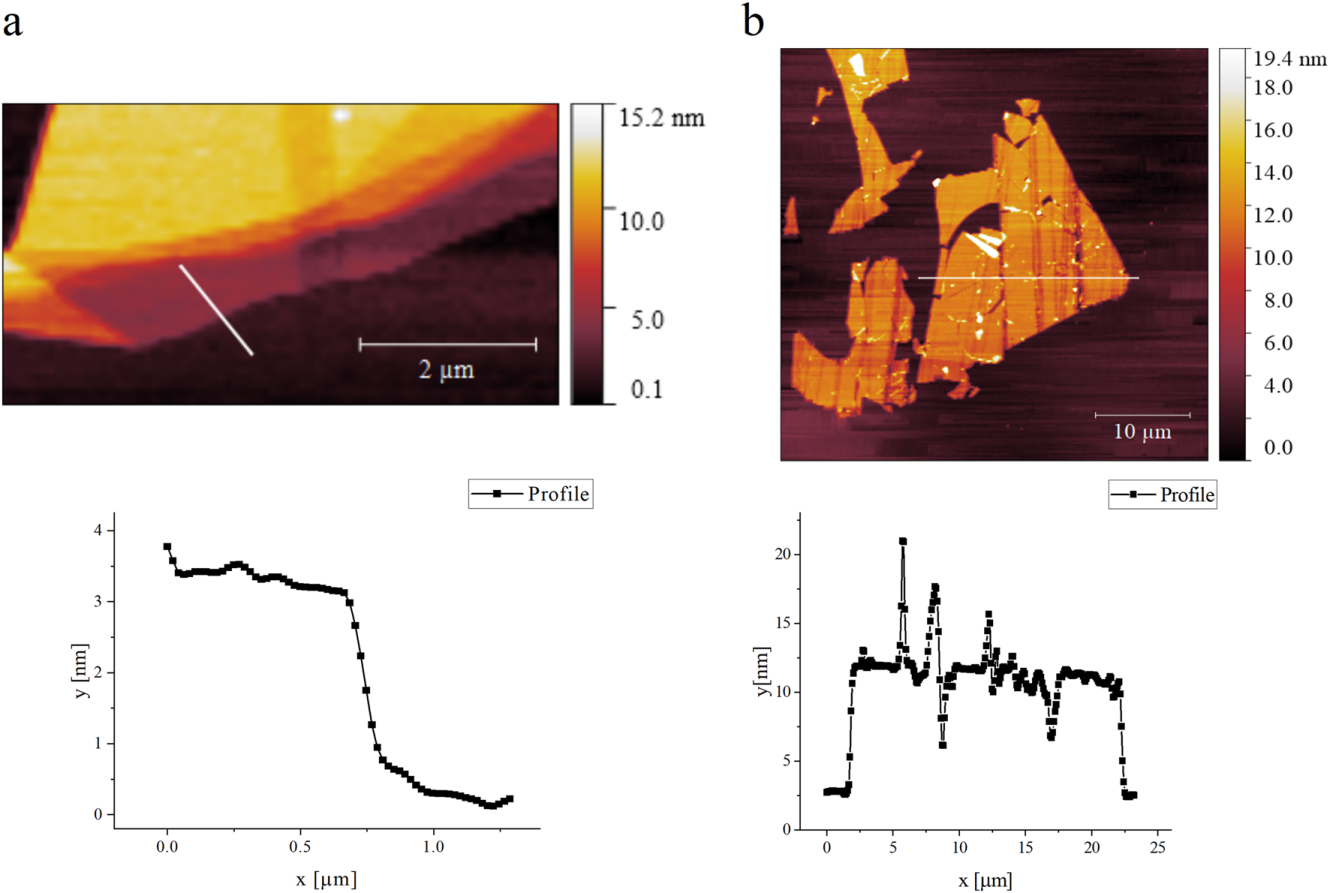
AFM topography images of muscovite mica on Au/Ti/Si substrate. (**a**) Tapping mode image for a 2–3 nm thick mica sheet with a thickness profile taken along the line indicated shown in the plot below. (**b**) Tapping mode image for a 9 nm thick mica sheet with a thickness profile taken along the line indicated shown in the plot below.

The thickness of the transferred mica flakes and local topography (both before and after electrical stressing) are characterized by tapping mode AFM. The production of large flakes is a feature of using the roller transfer method and AFM images show that many of the transferred flakes can be over 10 µm in size (Fig. 3b) with well-defined terraces providing extensive regions of constant thickness over which electrical measurements are undertaken. Thinner regions are often observed near the edge of the larger flakes, as shown in Fig. 3a. In this work, the thickness values over which measurements are taken range from 1.5 to 14 nm i.e., about 1ML to 14ML, where 1ML represents 1 mica layer.

**Figure 4:**
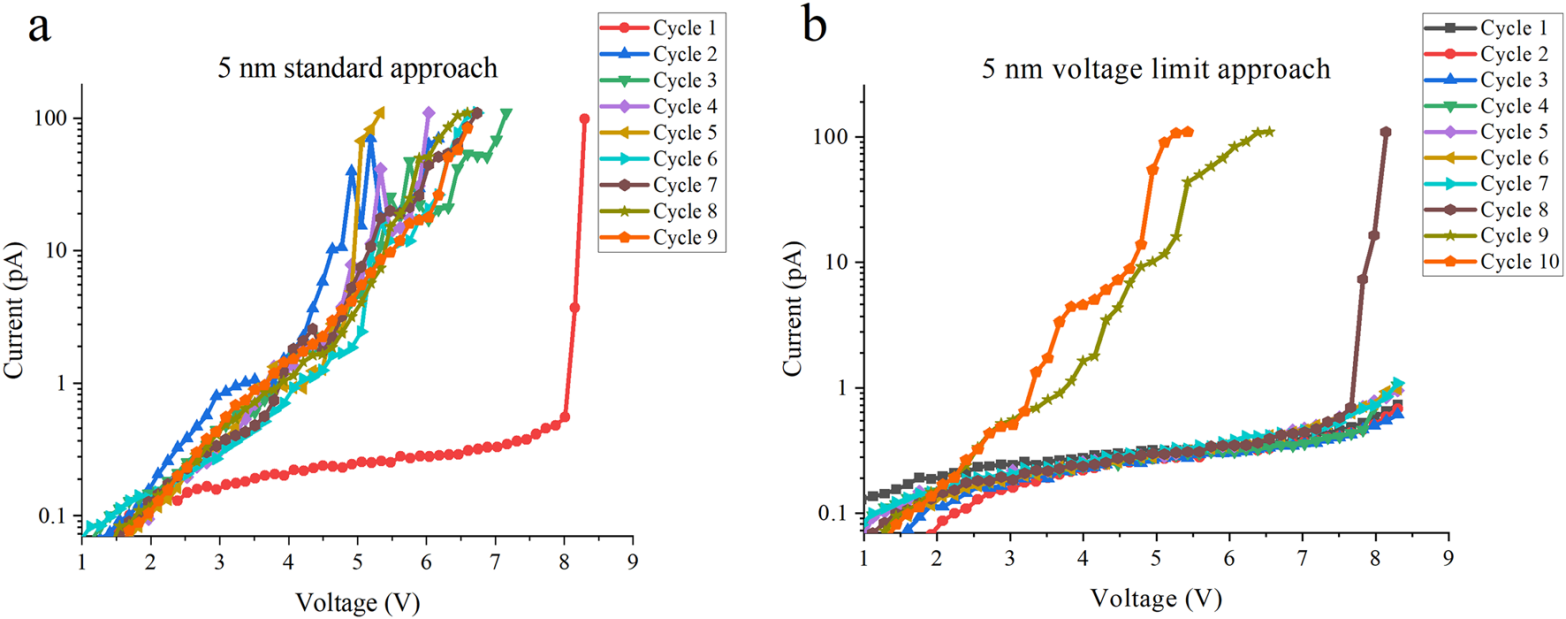
*I-V* curves for 5 nm flakes showing the two types of RVS measurement techniques. (**a**) Standard RVS with the voltage ramping until the current compliance (100 pA) is reached. (**b**) Voltage Limit RVS in which the voltage is ramped to slightly below the known BD value, in this case 8.3 V, allowing successive *I-V* to be undertaken before a breakdown occurs. A diamond cantilever is used for both data sets.

An important aspect of the electrical stress procedure is that two types of RVS are used, as shown in Fig. 4. In one procedure, the voltage is ramped up to a compliance limit with no restraint on the voltage (Fig. 4a). We refer to this as the *standard* approach. However, this method may lead to catastrophic damage to the mica and moreover the behavior of the current leading to breakdown (or equivalently, the charge injection) often cannot be adequately monitored because the current abruptly jumps from a negligibly small value to the current compliance i.e., the current spike at BD can mask small changes occurring just before BD. To monitor charge injection in the electrical breakdown process, we need to control and limit the current spike. We do so by ramping but then stopping the voltage slightly before the BD voltage (*V*_*BD*_), as shown in Fig. 4b. We call this the *voltage limit* approach, and it allows the mica to be continuously stressed prior to breakdown. In the example of Fig. 4b, the first seven cycles show a progressive increase in leakage current on successive stress cycles. At the  8th cycle , an abrupt change in current occurs which is associated with a BD in the material and subsequent cycles (5th and 6th) show considerably lower resistance as expected. The breakdown event itself is quite similar to that measured using the standard approach (Fig. 4a), although *V*_*BD*_ is a little smaller and the current change at BD is slightly more gradual rather than an abrupt current spike. Note that *V*_*BD*_ is defined at the current spike when the compliance limit is reached e.g., in Fig. 4a *V*_*BD*_ = 8.3 V at a compliance of 100 pA.

**Figure 5:**
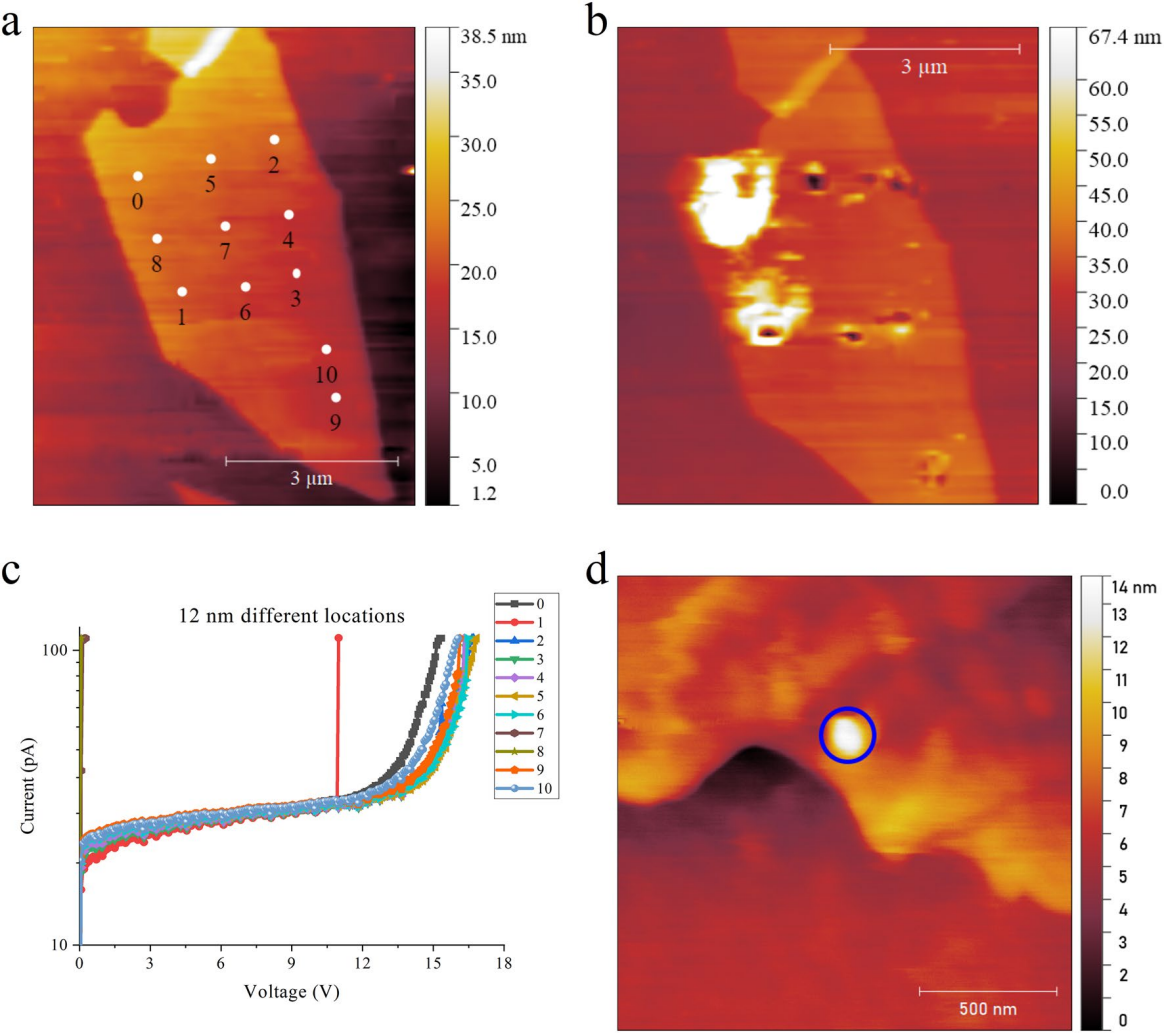
Experimental issues for CAFM measurement of mica dielectric breakdown. (**a**) Topography of a 12 nm mica flake before BD measurements. (**b**) Topography image of the flake after BD measurements using standard RVS with 100 pA compliance. Material damage (pitting) has occurred at the BD locations in this experiment, with the exception of locations 4 and 7. (**c**) The corresponding *I-V* curves taken during BD show that even though damage occurs, the post-BD locations are far enough apart as to not influence the measurement. The exception is location 8 which is between two large damage areas of Location 0 and 1. A PtIr cantilever is used. (**d**) Swelling (shown circled) on a 5 nm flake after a BD measurement using standard RVS with 100 pA compliance. The swelling is 8 nm in height.

We have found that there are three experimental issues to consider in obtaining consistent CAFM breakdown data, namely (A) an adequate distance between measurement locations must be maintained, (B) swelling of the mica under BD should be keep to a minimum, and (C) a low current compliance limit (~ 100 pA) must be set. These issues are summarized in Fig. 5. Figure 5a shows an AFM image of a pristine 12 nm flake indicating the locations for subsequent BD experiments. Figure 5b shows the same flake after the BD experiments using standard RVS. In this example, structural damage has occurred at all locations except points labelled 4 and 7. However, the associated BD *I-V* curves (Fig. 5c) indicate the breakdown measurement is consistent at all locations. The exception is point 8 which has a much lower *V*_*BD*_ and inspection of Fig. 5b shows that this measurement was undertaken between two previous BD experiments (points 0 and 1) which exhibited very extensive damage to the mica. These experiments indicate that for reliable data, measurements must be taken far enough apart (of order ~ 1 µm) so as not to influence any subsequent *I-V*. This requirement was also found for h-BN^31^, although the necessary separation was found to be smaller (~ 300 nm).

*Swelling* refers to the observation that after BD, a *hillock* forms at the BD location, as shown by the circled regions of Fig. 5d. The swelling only occurs if a current flows, although it is important to note that the current can be very low with swelling observed even at ~ 100 pA. Swelling has also been observed in h-BN but at significantly higher current level (~ 10–100 nA)^31,32^. The origins of the swelling in mica are still not known. Our conjecture is that the swelling is associated with the movement of intercalated potassium ions (K^+^) present in muscovite mica under the applied electric field^21^. However, dedicated experiments are needed in future to understand this phenomenon in-depth. From an experimental viewpoint, the *I-V* corresponding to the first BD should be reliable but subsequent *I-V* cycles should be treated with caution if excessive swelling is observed.

### Breakdown data on 2D mica

A key finding of our work is that the compliance current must be kept very low (~ 100 pA or less) for CAFM measurements on mica, both to prevent material damage and to allow the charge injection process to be monitored. We observe that increasing the current beyond ~ 100 pA typically leads to catastrophic damage of the material, even for very thin films (Supplementary Fig. S2). Obviously, one cannot obtain meaningful data on the post-breakdown characteristics (i.e., measure a 2nd BD *I-V*) over such damaged regions. For comparison, in CAFM experiments undertaken on similar thickness h-BN flakes, much higher currents can be reached before breakdown (see Supplementary Fig. S3), allowing the progressive degradation of the material to be readily observed under repeated RVS stressing at current limits up to ~ 100 nA^31^.

**Figure 6:**
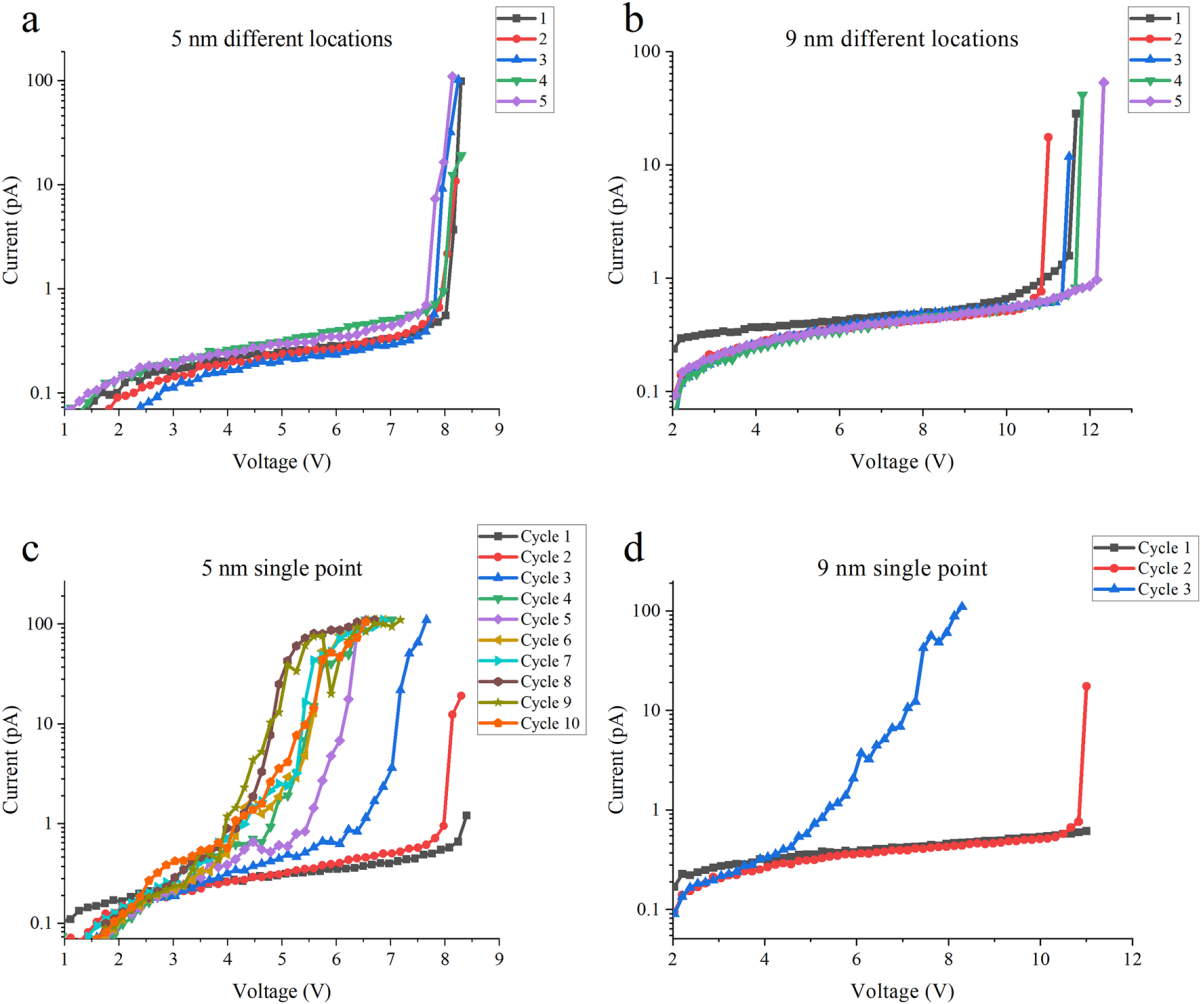
Typical breakdown *I-V*. BD measured by standard RVS across different points on (**a**) a 5 nm flake and (**b**) a 9 nm flake. There is only a small variation of *V*_*BD*_. Repeated stressing by voltage limited RVS measured at a single location on (**c**) 5 nm and (**d**) 9 nm flakes. Diamond cantilevers are used for all data.

Another major difference compared to h-BN is the breakdown event is very abrupt in mica when standard RVS stressing is used, as shown in Fig. 6a and b. Here, representative breakdown curves for different locations on various mica flakes are plotted and the curves show only minor variability for a given flake thickness. A key feature of all the *I-V* is that the leakage current is very low and the breakdown occurs abruptly. The leakage current through the mica flakes is less than ~ 1 pA at 3 V for all thicknesses measured (representing a current density of ~ 1 A cm^-2^ or less assuming a typical AFM tip-sample contact area of ~ 10 × 10 nm^2^^33^), even for 1–2 layers, indicating the good insulating properties of this 2D material. For comparison, the approximate thickness corresponding to a leakage current of 1A cm^-2^ at 1 V bias is 1.7 nm for SiO_2_^34^ and 1 nm for exfoliated h-BN^35^. There is a measurable increase in the current observed prior to BD as charge is injected into the mica, but the onset of the breakdown event itself is very abrupt. The effect of this small current flow is more readily observed in the repeated stressing of a single location using voltage limited RVS, as shown in Fig. 6c and d. In both figures, one observes current flow in Cycle 1, but no breakdown occurs. In Cycle 2, BD occurs, and subsequent *I-V* cycles show a degradation of the dielectric properties of the mica (i.e., lower resistance) under repeated stressing. The BD event is still abrupt but is measured at a slightly lower voltage than that using standard RVS stressing. Clearly charge injection is involved in the BD mechanism, as discussed in more detail below.

**Figure 7:**
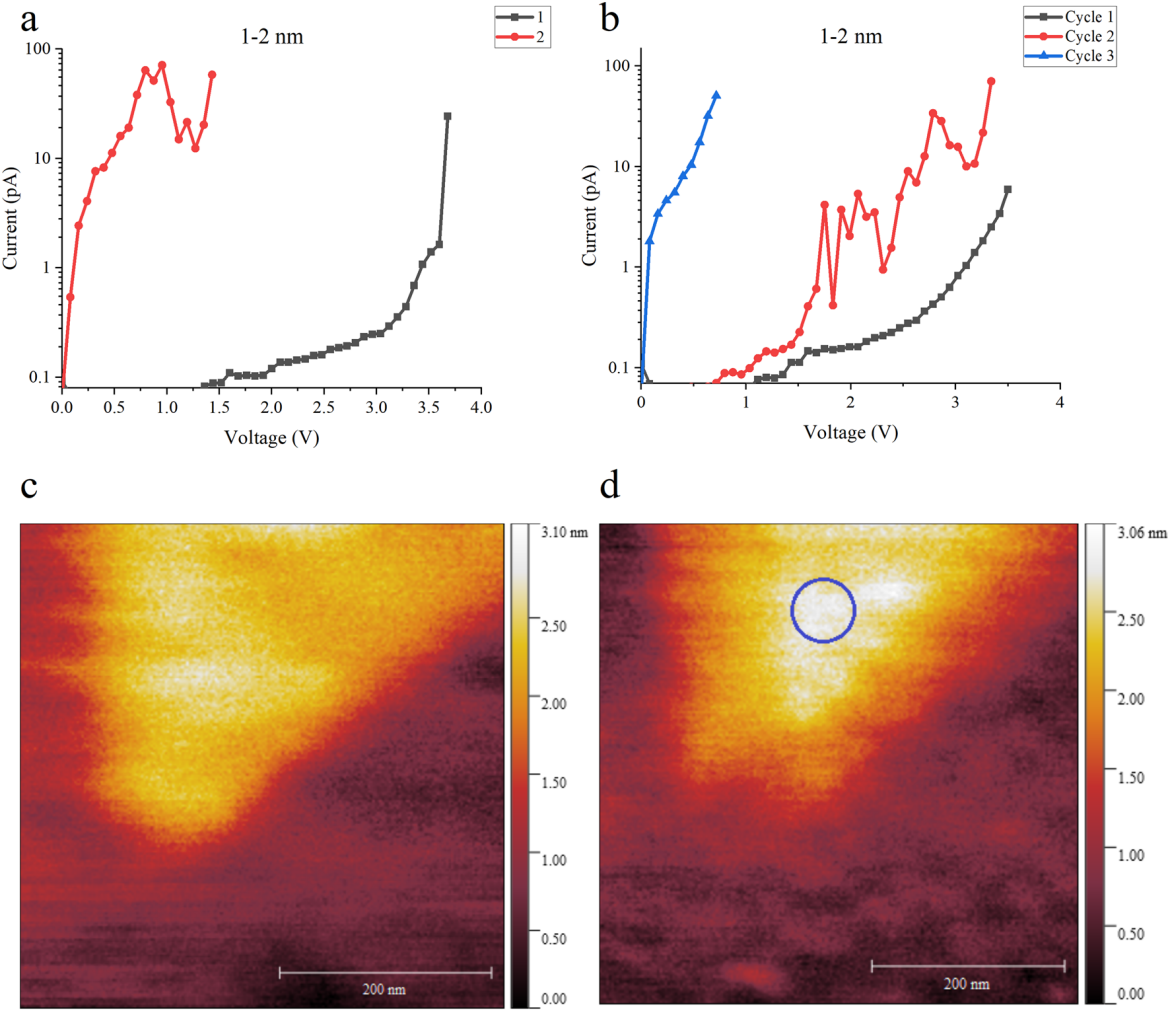
Breakdown data on very thin (1–2 nm) mica flakes, showing similar characteristics as for the thicker films. (**a**) BD experiment using standard RVS with 100 pA compliance, indicating *V*_*BD*_ = 3.6 V. A diamond cantilever is used. (**b**) BD experiment using voltage limit RVS up to 3.5 V with 100 pA compliance, indicating a progressive degradation stage in the BD process. A diamond cantilever is used. Topography of the BD region (**c**) before and (**d**) after the BD measurement showing a slight swelling (circled) at the BD location. The swelling is 0.8 nm high.

Interestingly, we observe the same breakdown trends even for very thin films, as shown in Fig. 7 for a 1–2 nm thick flake which we assign as being 2 layers (ML = 2). (Note: For very thin flakes, it is difficult to clearly define the thickness because of the roughness of the underlying gold). The BD is abrupt under standard RVS stressing (Fig. 7a) but shows a progressive degradation under voltage limited RVS with leakage current flow prior to the BD event (Fig. 7b). Subsequently, structural defects such as swelling may be observed at the BD location as shown in Fig. 7c and d. Irreversible damage can also occur if the current compliance is higher than ~ 100 pA (Supplementary Fig. S2). As above, these results indicate that increased current flow prior to BD is integral to the BD mechanism. In this regard, if repeated RVS stressing is undertaken to 1 V below the *V*_*BD*_, then no significant increase in current flow is observed and no BD event occurs.

**Figure 8:**
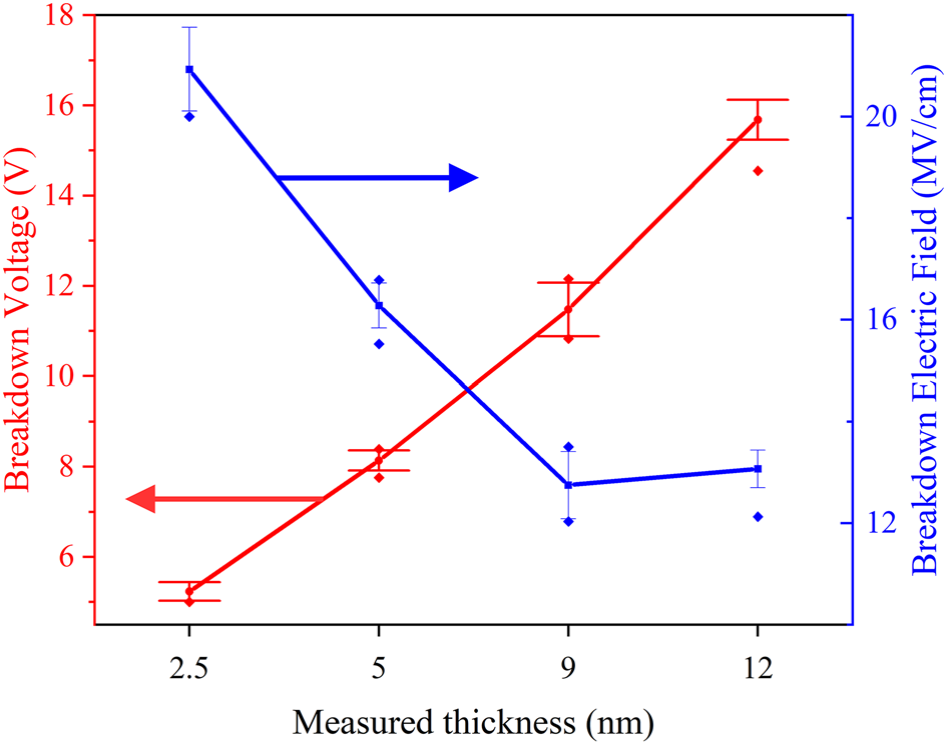
Plot showing the thickness dependence of *V*_*BD*_ and *E*_*BD*_, with the solid lines provided as a guide to the eye. The error bars indicate the spread of values measured with different tips and on different flakes.

The average *V*_*BD*_ for a given flake thickness can be found by measuring many *I-V* under standard RVS stressing e.g., *I-V* as in Fig. 6a and b. The electrical breakdown field strength (*E*_*BD*_) is then found as ${E}_{BD}= {V}_{BD}/{t}_{mica}$ where *t*_*mica*_ is the thickness of the flake. The average *V*_*BD*_ and *E*_*BD*_ values for flakes of different thicknesses is plotted in Fig. 8. It is apparent that the breakdown field is high, comparable to 2D h-BN^31^, and increases as the mica thickness decreases. This follows the same qualitative trend seen for amorphous dielectrics like SiO_2_^36,37^, the 2D dielectric h-BN^31^, and was also noted for thick (> 20 L) mica films^22^. Possible reasons for the thickness dependence include an increase in local electric fields for thicker layers due to an added contribution from material polarization^36,37^. Another possible reason could be an increase in the intrinsic defect density of thicker flakes.

## Discussion

### Comparison with previous experiments

There are only a few related studies reported for thin 2D mica^13,21,27,38^ using either muscovite mica or synthetic mica. The early work of Davidson and Yoffe^22,23^ studied BD in several natural micas and synthetic mica, although the samples tested were much thicker (> 20 ML) than recent studies. Importantly, they find both the BD and conductivity to depend on the type of mica investigated e.g., micas containing Fe and Ti have much higher conductivity. The more recent reports on ultra-thin mica investigate either muscovite or synthetic mica. Zou et al. studied the BD characteristics using a metal–insulator-semiconductor test capacitor, with 9 nm thick muscovite mica as the insulator layer^13^. A very abrupt BD is observed, just as in our work here, but the reported *V*_*BD*_ is lower (~ 8 V) compared to our measurement of *V*_*BD*_ ~ 11 V on 9 nm flakes. However, Davidson and Yoffe note that capacitor test structures yield noticeably lower *V*_*BD*_ than the comparable data taken with a gold metal wire probe^23^, because there are more inherent defects in the larger capacitor structure leading to higher currents at a given voltage compared to CAFM.

CAFM studies have also been reported on synthetic mica (Phlogopite mica)^27^ and muscovite mica^38^ with the *I-V* data presented showing currents up to 1nA and 100 nA, respectively. In our work, we cannot reach these current levels without BD or damage to the mica. Their voltage range is correspondingly also much lower than the voltages in our experiments e.g., for *I-V* measured on 3ML thickness mica, the current reaches 1 nA at just 3–4 V^27^ compared to *V*_*BD*_ ≈ 5–6 V in our work. The difference could be due to the use of different samples given the sensitivity of mica conductivity to mineral content^23^, or alternatively the structural difference between dioctahedral (muscovite) versus trioctahedral (phlogopite) i.e., trioctahedral silicate sheets have 3 divalent cations compared to 2 trivalent cations for dioctahedral silicate sheets. We suggest an alternative possibility, namely the mica has undergone breakdown. Neither of the studies^27,38^ mentioned if the data shown represents the first *I-V* or a subsequent *I-V*. The *I-V* reported could therefore reflect the conductivity of the material after BD. Note that the *I-V* after BD still appears non-linear (Supplementary Fig. S4) but the current for a given voltage is much higher.

The CAFM studies using 2D synthetic mica (Fluorophlogopite mica) as the active material in an RRAM device^21^ provide further support to our observations. Here, a forming voltage is applied across the mica over many RVS cycles to achieve a stable RRAM response. This use of forming voltage is entirely similar to our data taken with a voltage limit RVS. If one carefully considers the *I-V* data shown for the forming voltage cycles, one can discern that the first *I-V* is similar to our BD data with the following voltage cycles showing decreasing resistance until the stable RRAM configuration is reached.

### Breakdown characteristics of 2D mica

The primary insulating material considered for 2D applications is h-BN and hence it is worthwhile to compare the mica and h-BN breakdown characteristics. The leakage current values measured by CAFM are quite similar (< 1 pA over comparable thickness and measurement ranges) for both mica and h-BN^39^ and *E*_*BD*_ increases as the film thickness decreases for both materials^31^. A significant difference is the abrupt, catastrophic breakdown for mica at very low currents (< 1 nA), whereas the BD in h-BN occurs at much higher current compliance (> 100nA) and a progressive layer-by-layer degradation of the dielectric is measured^31,40,41^, as shown in Supplementary Fig. S3. Catastrophic BD was observed even for 3ML mica films.

This stark contrast in BD behavior between mica and h-BN implies there must be a fundamental difference in the BD energetics. However, an understanding of the BD mechanism at the atomic scale is lacking with further experimental and computational studies required. For example, studies on synthetic mica have shown that intercalated potassium ions under an electric field migrate vertically through the mica layers^21^. In our work, *swelling* resulting from the BD measurement is observed at the tip location and we surmise this arises from the electric field driven movement of K^+^ ions. Importantly, swelling only occurs if a relatively significant leakage current is detected *just* prior to BD, suggesting the movements of K^+^ ions *may be* significant only during the BD transient. However, the nature or even existence of a BD mechanism involving K^+^ remains unknown.

Hence, we limit the discussion to a phenomenological description based on the large body of work available for conventional thin film oxides^42^ and 2D materials^43^. Broadly, we describe why the BD only occurs at very low current levels and why the BD always appears abrupt and hence, progressive degradation is not observed.

In a conventional gate dielectric such as SiO_2_ or HfO_2_, there is an initial wear out phase, followed by the onset of BD in which a current carrying leakage path linking the two electrodes is formed i.e., a percolation path^42^. In our work, a small rise in current is observed in the wear out phase prior to BD and as in other dielectrics, this charge injection gives rise to an accumulation of defects leading to the BD itself^23,42^. We find charge injection occurs prior to BD for both muscovite mica and synthetic mica (Supplementary Fig. S5). In contrast, whilst Davidson and Yoffe also find current flow prior to BD for natural micas^22,23^, they observe no measurable current for synthetic mica. We attribute this difference to the much thinner films used in our work. No discrete or digital fluctuations in the current can be observed, but this is to be expected given the very low current levels that must be measured with a correspondingly small measurement bandwidth (~ 1.2 Hz).

The wear out and BD are followed by a third phase in which material damage occurs as rapid changes in power dissipation and local temperature enhancement around the percolation path take place at the BD location. It is the time evolution of this third phase which dictates the experimental observations for mica. Specifically, it has been shown that after the percolation path is formed, the rate of change of the breakdown current (*dI*_*BD*_*/dt*) is a key parameter in the BD evolution^42^. For thick films (> 10 nm), which necessarily also corresponds to high voltages, the BD transient *dI*_*BD*_*/dt* is large. Consequently, for SiO_2_ and HfO_2_, the BD evolves extremely rapidly (at nanosecond time scales) and when measured using typical instrumentation at the millisecond time scale, always appears as a hard abrupt breakdown. As the film thickness is reduced, and thereby also the corresponding voltage, the kinetics of the BD evolution slows down drastically such that a gradual growth in current may be observed. This is termed progressive BD in SiO_2_ and HfO_2_ (see Supplementary Fig. S3) which occurs not only because the stress voltage is less but also because the injected electron energy is smaller such that specific breakdown processes cannot be activated^42^. In fact, it has been argued that a progressive BD will always occur in a material, but often the effect is masked as the experimental measurement latency is too slow to catch up^42^. For SiO_2_, the BD transient and related kinetics can be associated with the critical defect density necessary for breakdown (*N*_*BD*_), and the dependence of *N*_*BD*_ with SiO_2_ thickness is well known^44^. For example, the larger *N*_*BD*_ of thick films results in abrupt, fast BD because the large number of defects present near the percolation path means that current can flow along many available paths linking the two electrodes^43^. Further study needs to be done to verify if this phenomenon also applies to mica.

In our experiments on mica, hard BD is invariably observed, even for very thin films (2ML) at low voltage, indicating *dI*_*BD*_*/dt* is high and the BD is very fast. Any progressive (i.e., slower) BD features are masked, although there are traces of progressive breakdown in some of the measurements on thinner samples under voltage limited RVS e.g., Figs. 6c and 7b. However, these should be considered only as tentative indicators and further investigation, specifically time-dependent-dielectric-breakdown (TDDB) experiments, are required. The most likely reason *dI*_*BD*_*/dt* is always high stems from the poor thermal conductivity of mica (k_th_ ~ 1 Wm^−1^ K^−1^). For comparison, 2D h-BN has k_th_ ≥ 100 Wm^−1^ K^−1^^45^. Once a percolation path has formed and more current begins to flow through the BD location, the power generated at the BD increases substantially. In low thermal conductivity materials, the heat cannot be efficiently dissipated and, aided by thermal runaway, the BD spot can rapidly reach very high temperature (> 1000 °C)^43^ leading to structural damage. The poor heat dissipation also implies that once a BD path has formed, only a very low current is required to initiate thermally induced damage. This appears to be the case for mica because catastrophic BD damage occurs even at ~ 1 nA current compliance as measured by CAFM. Note that a direct comparison of thermal BD effects between mica and conventional gate oxides, which also have poor thermal conductivity (e.g. k_th_ ≈ 1 Wm^−1^ K^−1^ for SiO_2_^46^), is complicated by the fundamental structural difference between the materials i.e. SiO_2_ and HfO_2_ are amorphous, whereas mica and h-BN are layered crystalline materials. Hence, at present, it is only meaningful to compare the mica data with h-BN. Clearly, further studies such as density functional theory, molecular dynamics simulations, and high resolution transmission electron microscopy are essential to elucidate the BD mechanism at the atomic scale.

## Conclusion

We have performed localized electrical breakdown measurements on 2D muscovite mica flakes of ~ 2 to 15 nm thickness by CAFM. This is the first dedicated study of mica breakdown since the seminal work of Davidson and Yoffe^22,23^ on thick (> 20 nm) mica sheets. For CAFM studies, we have found that there are three important issues to address to obtain robust BD data. First, the BD locations must be spaced sufficiently far apart (> 1 µm) to ensure that there is no interference between measurements. Second, the current compliance must be set to a very low value (< 1 nA) to ensure severe damage does not occur to the sample in a BD event. Finally, swelling of the mica occurs once a current begins to flow. The origins of the swelling are not understood at present (we surmise it could be due to the field driven diffusion/ electro-thermo migration of potassium ions) but from an experimental viewpoint, the BD data should be treated with caution for locations where excessive swelling is observed.

We find that 2D muscovite mica has a high electrical breakdown strength (12 MV/cm or more) and low leakage current, comparable to 2D h-BN of similar thickness. However, a significant difference compared to h-BN and conventional thin film dielectrics is the very small current compliance necessary to avoid catastrophic damage in a BD event, even for very thin (2-3ML) flakes. The resulting BD *I-V* data always appears very abrupt and no progressive BD process can be definitively observed, although a measurable current flow is required prior to initiation of the BD event (presumably due to the generation of defects). No atomistic understanding is available at present for mica BD and we suggest a general reason for the marked differences between mica and h-BN to arise from the poor thermal conductivity (two orders of magnitude lower than h-BN) of mica. As power cannot be effectively dissipated, the temperature rise at the BD location can be rapid and large, leading to thermally induced damage even at very low currents.

## Methods

We use V1 grade muscovite natural mica (50 mm × 75 mm × 0.15 mm substrates) from Ted Pella, Inc. and Fluorophlogopite synthetic mica (20 mm × 10 mm × 0.2 mm substrates) from Changchun City Taiyuan Fluorophlogopite Co. Ltd. for preparing natural mica and synthetic mica flakes, respectively. Gold assisted mechanical exfoliation of mica is used (Supplementary Fig. S1) with the mechanical exfoliation of the bulk mica performed using a polyurethane gel roller of diameter 20 mm (EX-230-AS, EXSEAL, Co, Ltd, Mino, Japan)^27^. The resulting 2D mica layers attached to the roller are immediately transferred onto a freshly deposited gold surface under clean room conditions. The gold film is 20 nm thick with a 2 nm titanium adhesion layer and is deposited on a silicon substrate by electron beam evaporation (Denton Explorer).

The resulting flakes on the gold surface are first imaged optically and then imaged using an Atomic Force Microscope (AFM). The AFM system used (JPK NanoWizard 3) is first operated in tapping mode (using Tap190-G, k_c_ = 48 N/m cantilevers from Budget Sensors, with k_c_ being the cantilever stiffness) to obtain high resolution images of the flakes. Once suitable flakes are identified, CAFM is used to perform localized BD on the flakes by RVS. A Keithley 4200 Semiconductor Characterization System is used for RVS to force voltage onto the tip and measure the current passing through the tip (Fig. 2). The gold substrate is grounded. We find that to avoid catastrophic electrical damage of the mica, the Keithley 4200 must operate at a low current measurement range (1nA) and very low current compliance (typically < 0.1 nA).

Two types of tips are used for CAFM, namely Platinum Iridium wire cantilevers (Rocky Mountain Nanotechnology 25PtIr300B, k_c_ = 22 N/m) and conducting diamond cantilevers (Budget Sensors CDT-FMR, k_c_ = 6.2 N/m). The tip resistance is constantly checked during CAFM measurements by moving off a mica flake and measuring the *I-V* on the nearby gold surface. A representative *I-V* taken on gold is shown in Supplementary Fig. 6. This check is important for CAFM on insulating samples such as mica to ensure the tip apex maintains low resistance electrical characteristics and is not damaged or contaminated. CAFM measurements are taken in ambient conditions under flowing nitrogen to decrease the relative humidity to ~ 20% and only positive bias is applied to the tip; both arrangements are to mitigate effects of local anodic oxidation at the tip^47,48^. Finally, it is worth noting that the total measurement time for each point selected takes less than a minute and hence, with the cantilevers used, the lateral drift of the tip location is not significant for the measurements presented^49^.

## Supplementary Information

Supplementary Information.

## Data Availability

The data that support the findings of this study are available from the corresponding author upon reasonable request.
