# Supplementary material for: Dielectric breakdown of 2D muscovite mica
Source: Sci Rep. 2022 Aug 18;12:14076. doi: 10.1038/s41598-022-18320-7 (PMC9388672; doi:10.1038/s41598-022-18320-7)
Supplement: Supplementary file 1 — Supplementary Information. [file 41598_2022_18320_MOESM1_ESM.pdf]

Supplementary Data

Dielectric Breakdown of 2D Muscovite Mica

Anirudh Maruvada, Kalya Shubhakar, Nagarajan Raghavan, Kin Leong Pey & Sean J. O'Shea

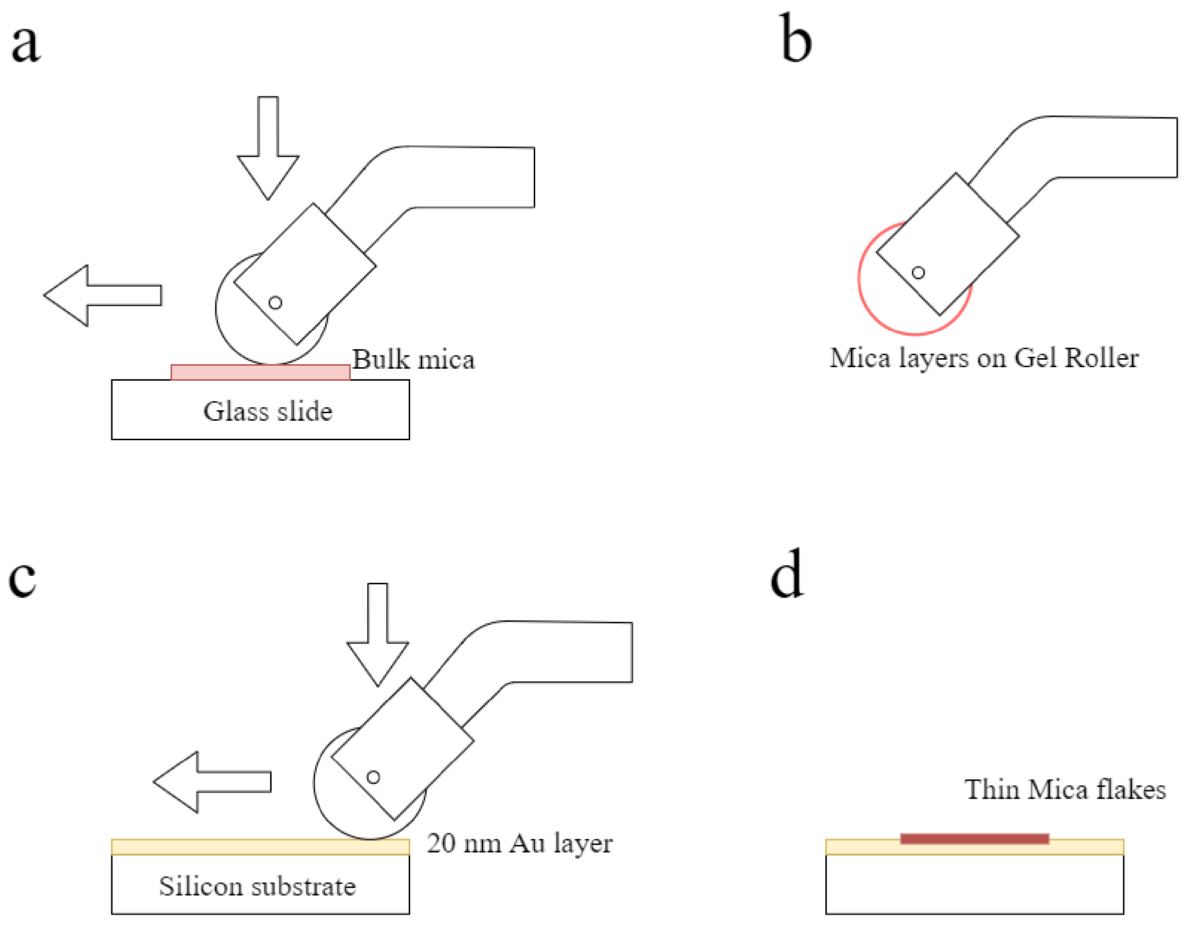

Figure S1. Schematic representation of mechanical exfoliation and transfer process [1]. a,b) Mica layers are exfoliated by the rolling application of a polyurethane Gel Roller (Exseal Corporation Semiconductive, Japan, gel roller EX230-AS, diameter 20 mm, width 30 mm). c,d) The roller is then used to transfer the mica flakes onto the gold substrate

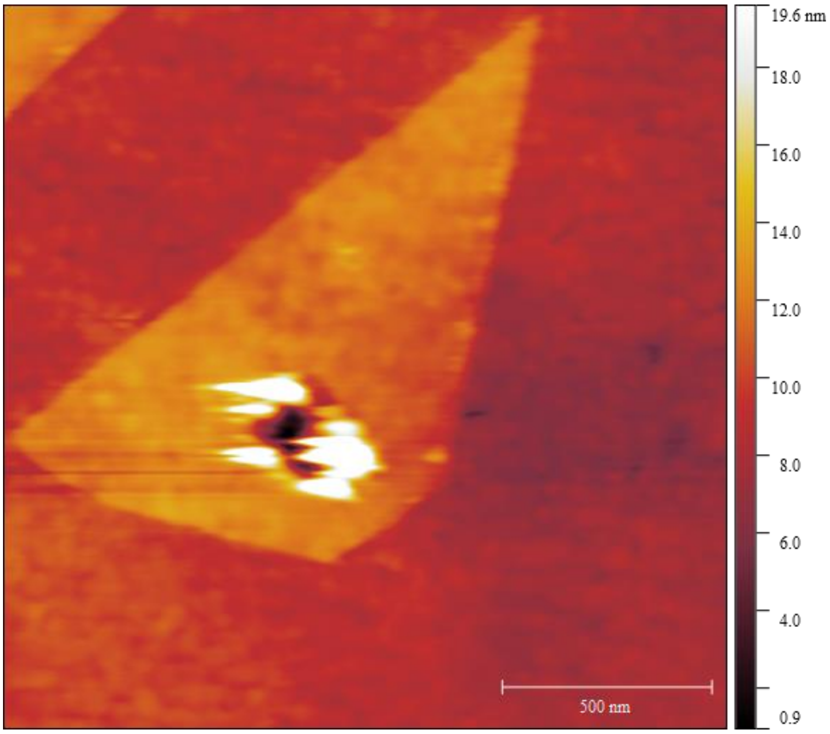

Figure S2. Topography of a 3 nm thick flake after a BD experiment using 1 nA compliance. The BD has damaged the flake even though it is very thin.

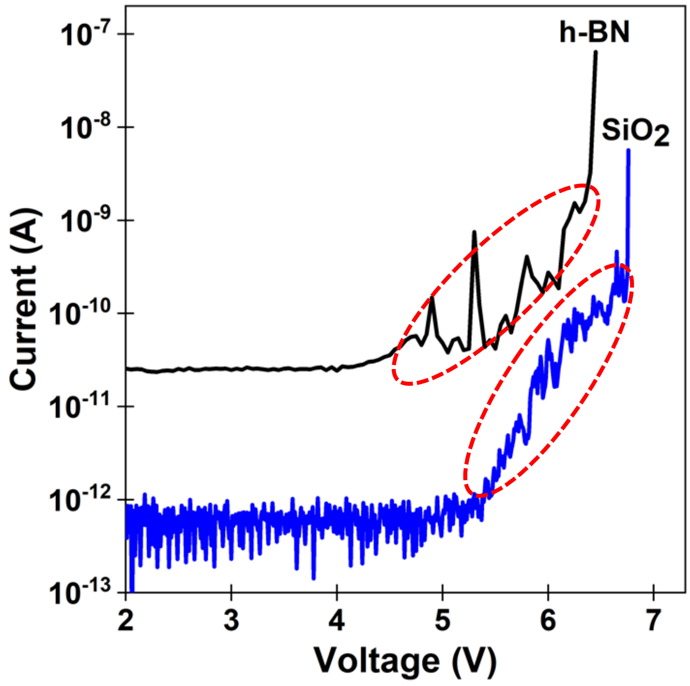

Figure S3. *I*-*V* curves taken by RVS using CAFM showing breakdown measured on 3 nm thick h-BN on graphite and 3.3 nm thick SiO_2 on Silicon. A PtIr and Pt wire cantilever have been used for measurements on h-BN and SiO_2 respectively. The dotted regions indicate progressive degradation of the material prior to the sudden onset of breakdown. Note, the order of magnitude higher leakage current at low voltage for h-BN is an experimental artefact because the lowest current detection level is set by the compliance range chosen, which is 100 nA for h-BN and 10 nA for SiO_2 in this data set. Reproduced from [2] with permission.

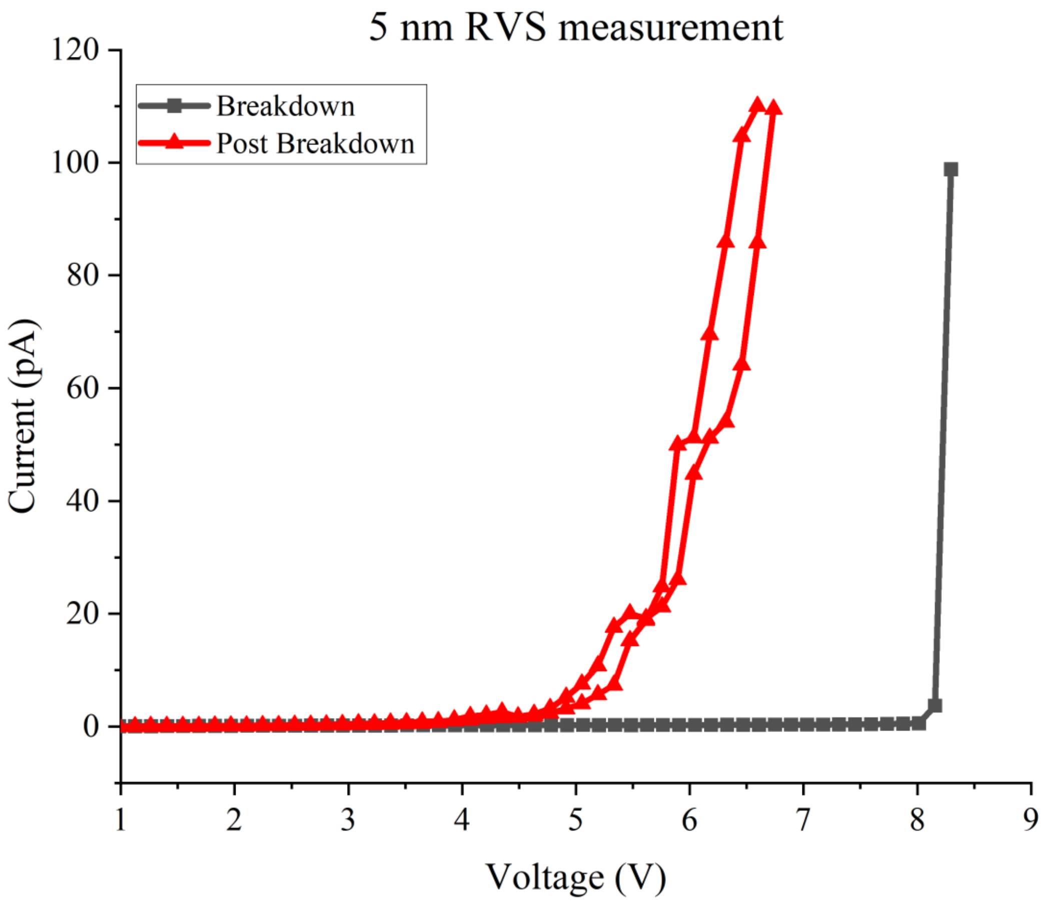

Figure S4. The data of Figure 4a taken on a 5 nm flake re-plotted on a linear current scale. A diamond cantilever is used. One observes the post-BD curves appear (approximately) exponential as also reported in previous works measuring *I-V* by CAFM. This may indicate some previously reported data could represent mica that has undergone a BD rather than pristine mica.

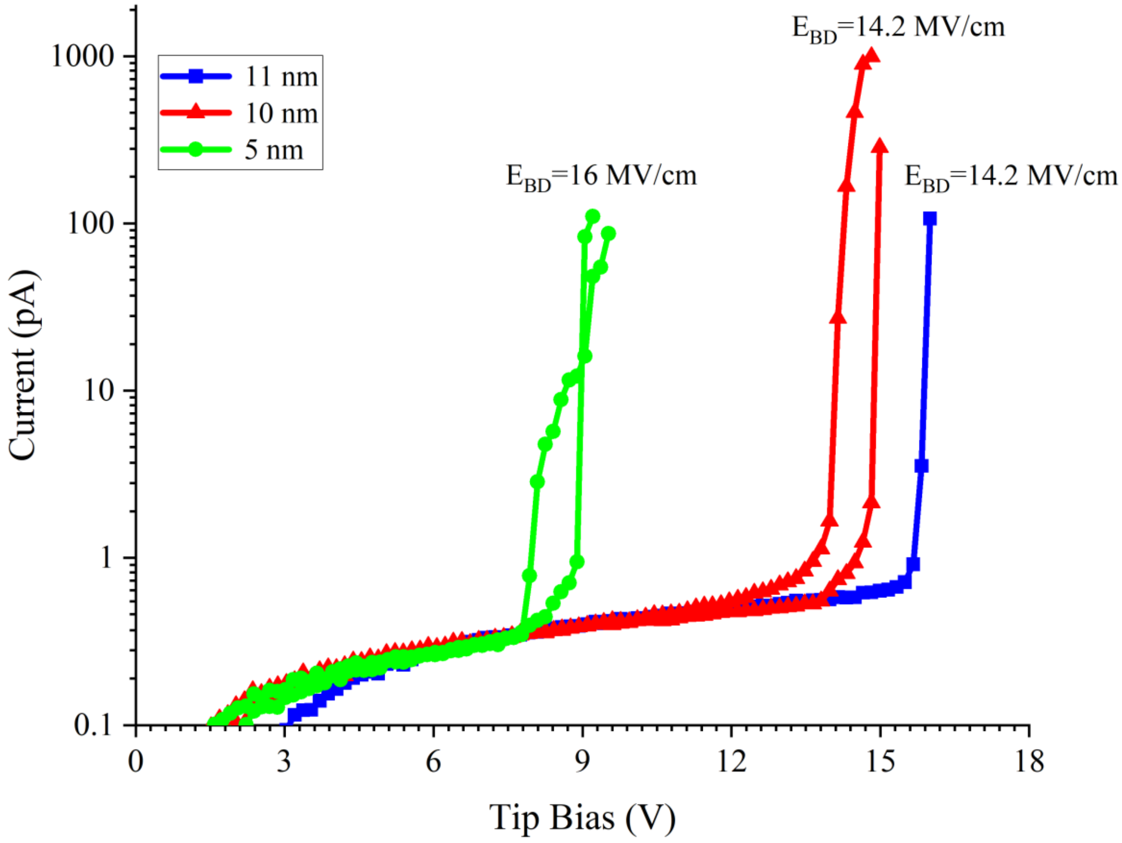

Figure S5. Preliminary BD measurements using standard RVS taken on synthetic mica. The curves are colour classified by their flake thickness with the estimated E_{BD} shown. Synthetic mica is also sensitive to high current and therefore a low current compliance (0.1 – 1 nA) was set. A diamond cantilever is used for the 5 nm data and a PtIr cantilever for the 10 nm and 11 nm data. The BD behaviour of synthetic mica appears essentially the same as for natural mica i.e. the leakage current is low and the BD event is abrupt. The E_{BD} for 5 nm synthetic mica is similar to muscovite mica but is slightly larger (14 MV/cm versus 13 MV/cm) for the 10-11 nm thick flakes.

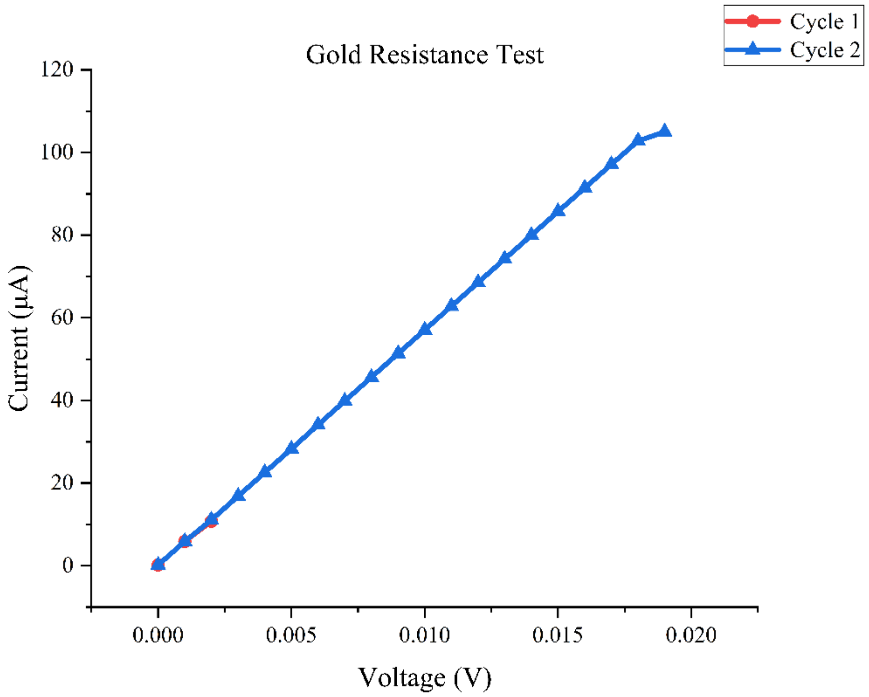

Figure S6. Representative *I*-*V* curve taken with a PtIr wire cantilever on the gold surface to test the resistance of the AFM tip during electrical breakdown measurements. The *I*-*V* trend is ohmic and the resistance is 180 Ω, indicating the AFM tip has good conductivity for CAFM.

References for Supplementary

[1] M. R. Islam and M. Tomitori, Evaluation of the discrete thickness of exfoliated artificially synthesized mica nanosheets on silicon substrates: Toward characterization of the tunneling current through the nanosheets, *Applied Surface Science*, **532**, 147388, doi: 10.1016/j.apsusc.2020.147388 (2020).

[2] A. Ranjan, *et al*. Dielectric Breakdown in Single-Crystal Hexagonal Boron Nitride, *ACS Applied Electronic Materials* **3**, 3547-3554, doi:10.1021/acsaelm.1c00469 (2021).
